# Rethinking asthma therapy, part 1: transdermal delivery of β_2_-agonists

**DOI:** 10.3389/jpps.2026.15713

**Published:** 2026-03-04

**Authors:** Joseph Correa, Nicole K. Brogden

**Affiliations:** 1 Department of Pharmaceutical Sciences and Experimental Therapeutics, University of Iowa, Iowa City, IA, United States; 2 Department of Dermatology, University of Iowa, Iowa City, IA, United States

**Keywords:** asthma, beta agonist, drug delivery, skin, transdermal

## Abstract

Asthma and allergies are closely related conditions affecting millions of people around the world. Current treatment options cover many classes of drugs for both acute and ongoing conditions. β2-agonists, leukotriene modifiers, and corticosteroids represent some of the common types of drugs utilized in asthma management. These medications are often delivered via inhalation, oral, or parenteral methods, but each of these modalities faces challenges due to improper technique with inhalers, lessened oral bioavailability due to first-pass metabolism, and reduced compliance of injectable medicines. Transdermal drug delivery may offer a beneficial route of administration that overcomes these barriers as a painless, self-administered form that bypasses first-pass metabolism and can reduce dosing frequency with longer drug release profiles and reduced fluctuations in plasma drug levels. In this two-part mini-review series we will summarize the current literature on transdermal systems for asthma and allergy therapy. Here in Part 1, we cover β2-agonists and discuss the potential of transdermal systems for these drugs. While the body of work with transdermal β2-agonists for asthma treatment is limited, there is still evidence that transdermal systems for asthma has potential to greatly shift the field of asthma therapeutics.

## Introduction

Asthma is a well-described and highly complex disorder involving chronic airway inflammation that results in hyperreactivity, coughing, and chest tightness. This disorder impacts more than 300 million people globally [1]. Beyond the physical detriments of asthma, there are many financial burdens associated with treatment and other healthcare costs. Across Europe, the average direct cost of asthma per patient in 2019 was approximately €2,200 (values were reported for the 2021 Euro, converting to ∼$1,900 2021 USD without inflation adjustment) [2]. Among working adults in the United States from 2018–2020, annualized asthma-related medical costs were approximately $2,600 per person with total incremental medical costs (costs beyond regular expenses) and total medical costs of $3.8 billion and $21 billion (2022 USD), respectively [3].

Asthma variants include allergic, non-allergic, occupational, exercise-induced, cough variant, aspirin-exacerbated, nocturnal, and asthma-chronic obstructive pulmonary disease overlap [1, 4–8]. Of these forms, the two main subtypes are allergic and non-allergic [4, 5]. Asthma and allergies are often closely related (allergens can stimulate asthmatic reactions/symptoms in allergic asthma) and >80% of asthma patients also have allergies [6, 8, 9]. While allergic and non-allergic asthma reactions occur in response to different triggers (e.g., pollens or mold for allergic and air pollution or respiratory infections for non-allergic), these classifications are not mutually exclusive, and patients with allergic asthma will often react to the same non-allergic triggers as patients with non-allergic asthma [7, 8]. Treatment options are similar for both subtypes: pharmacological treatments and avoiding triggers, and the introduction of monoclonal antibodies provides an additional treatment modality [5, 9, 10], which may be especially important for allergic asthma because immunotherapies and biologic treatments are more individualized and specific to this subtype [7].

## Pharmacologic asthma treatment

Regardless of subtype, asthma treatment goals are multi-fold, including symptom control, reducing risk of exacerbations, and minimizing medication adverse effects [4, 5, 7]. Treatment is typically divided into rescue and maintenance therapies. For acute episodes, fast-acting (“rescue”) treatments are used, which include inhaled short-acting β2-agonists and oral or intravenous corticosteroids [1]. Intravenous magnesium and subcutaneous epinephrine may be used in more serious cases [1]. Maintenance therapies aim to proactively prevent or reduce the likelihood and frequency of asthma episodes or flare-ups. Standard of care maintenance therapies include long-acting β2-agonists, inhaled corticosteroids (ICS), and leukotriene modifiers [5, 8]. For chronic asthma, combination therapies of inhaled long-acting β2-agonists and increasing doses of ICS are commonly implemented [11]. If a patient does not respond to this combination, long-acting muscarinic antagonists may be added [11]. Additional adjunct therapies include (but are not limited to) oral corticosteroids, theophylline, mast cell stabilizers, and monoclonal antibodies [7–11].

## Benefits of transdermal drug delivery

**FIGURE 1:**
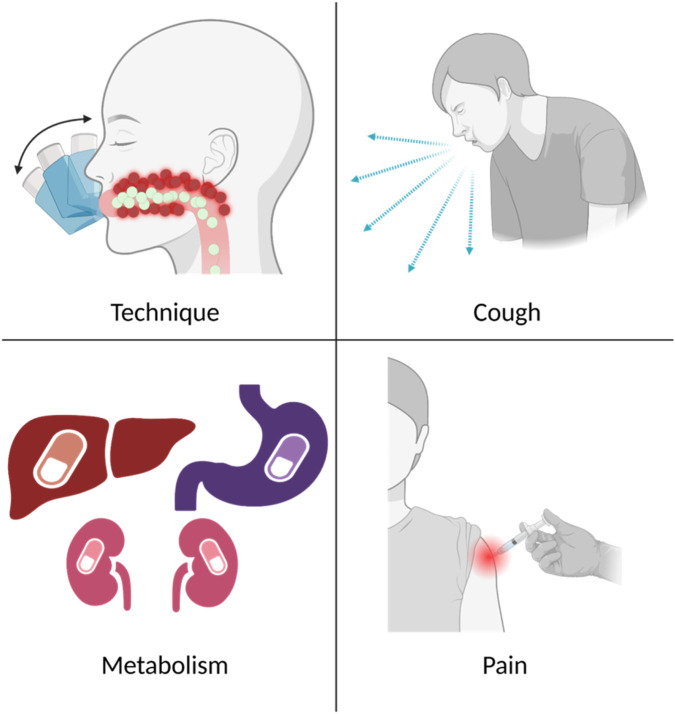
Common problems associated with currently available treatment for asthma. Top left, improper inhaler techniques causing drug particles to impact in the mouth or upper airways. Top right, coughing, in response to inhaled medication, ejecting drug from the lungs. Bottom left, metabolism and breakdown of the drug in the stomach, liver, or kidneys. Bottom right, pain with injections. Created in BioRender. Correa, J. (2026) https://BioRender.com/vejmfo0.

Asthma treatments are primarily delivered via inhaled or oral dosage forms, though injectable or intravenous therapies may also be necessary. While inhaled medications are often preferred for respiratory diseases, there are challenges affecting drug disposition in the respiratory tract including improper inhaler technique or coughing resulting from inhaled medications, leading to lowered medication efficacy (Figure 1) [12–17].

As a potential alternate dosage form, transdermal delivery could present benefits for asthma and allergy management. Drug absorption through skin into the systemic blood supply is non-invasive, painless, and self-administered [18]. First-pass hepatic metabolism (often associated with oral dosage forms) is bypassed, and plasma concentrations are more consistent over the full dosing period, avoiding large peaks and troughs [19]. Transdermal delivery also provides a longer drug release profile, often reducing dosing frequency; this can be a notable benefit for drugs with short half-lives that require repeated dosing [20, 21]. Collectively, these benefits can increase patient compliance and positively impact efficacy [18].

Despite potential benefits, exploration of transdermal dosage forms for asthma and allergic conditions has been relatively limited. The goal of this two-part mini-review series is to broadly summarize the current literature regarding advances in transdermal treatments for asthma and allergies. In Part 1 we summarize studies of transdermal short- and long-acting β2-agonists. In Part 2, we focus on transdermal delivery of other drug classes used for asthma, including newer and emerging therapies, and transdermal treatments for general allergic conditions (asthma and allergies often co-exist, making a brief discussion of allergy treatments pertinent to the overall topic). While there are not major differences in the drug classes used to manage adult vs. pediatric patients with asthma and allergies, our reviews focus on FDA-approved treatments for patients >12 years of age.

## Studies of transdermal β2-agonists

β2-agonists are commonly prescribed to treat and manage asthma because of bronchodilatory effects that improve airflow. β2-agonists are classified as short- or long-acting, depending on duration of symptom management and place in therapy.

### Short-acting β2-agonists (SABAs)

SABAs are commonly used as rescue (“as needed”) therapies for acute episodes in all asthma subtypes, or to manage intermittent bronchospasm [1, 5, 11]. Typically delivered as inhalations, frequent administration is required–ranging from every 20 min to every 4 – 6 h [1, 5, 22, 23]. A sustained SABA release profile would be favorable to reduce this need for repeated dosing [24, 25], and studies of transdermal SABA formulations have achieved sustained and controlled delivery to maintain therapeutic levels [25–28], Table 1.

**TABLE 1:** Short-acting β2-agonists.

Experimental technique	Drug and formulation	Endpoint	Results	Ref.
*In vitro*/*ex vivo*	Salbutamol (5 mg) + 2% HPMC + 2% IPM	Cumulative amount permeated across human skin over 24 h	2,400 μg	[29]
		Flux across human skin	108.89 ± 5.83 μg/h from 10 cm^2^ patch	
	Salbutamol + Myverol 18–99	Amount transported across dialysis membrane over 2 h	11 mg/mL dose: 1 mg/cm^2^ 27 mg/mL dose: 2.7 mg/cm^2^ 46 mg/mL dose: 3.7 mg/cm^2^	[30]
		Flux across murine skin	Passive: 1.12 × 10^-3^ mg/cm^2^ min Iontophoretic: 5.4 × 10^-3^ mg/cm^2^ min	[31, 32]
	2% salbutamol + 0.5% HPC + lauric acid (2:1 or 3:1 to salbutamol)	Flux across human skin	2:1 ratio: 0.089 ± 0.003 mg/cm^2^ h 3:1 ratio: 0.050 ± 0.004 mg/cm^2^ h	[33]
	16% salbutamol + 71.9% silicone + 10% n-dodecanol + 1.75% glycerol + 0.35% hexanol	Cumulative amount dissolved in water in Hanson dissolution apparatus	Single layer: 2.34 ± 0.14 mg/cm^2^/day Double layer: 2.37 ± 0.25 mg/cm^2^/day	[27, 34]
	Salbutamol + HEMA/DMAPMA (50/50)	Cumulative release	30% over 30 h	[35]
	0.01 M salbutamol + 0.01 M methyl-orange ion pair	Iontophoretic flux across rabbit skin	pH 5: 0.45 μg/cm^2^ h pH 7: 0.52 μg/cm^2^ h	[36]
	2% salbutamol-loaded cubosomal gel	Cumulative percent permeated across rat skin	1.5 g MO: 95% in 24 h 2.5 g MO: 95% in 72 h 3.5 g MO: 70% in 72 h	[25]
	10 mg salbutamol in ethosomes	Cumulative percent permeated across mouse skin	20% EtOH: 23.15% 30% EtOH: 27.32% 40% EtOH: 28.12%	[37]
	6 mg salbutamol + 1.5 mg ketoprofen in Eudragit RL 100 matrix dispersion with plasticizer and enhancers	Cumulative amount of salbutamol permeated across rabbit skin over 24 h	PG: 2.747 mg Tween 20: 3.949 mg IPM: 4.235 mg Eucalyptus oil: 4.121 mg Castor oil: 2.787 mg Span 20: 4.144 mg	[24]
	2.5 mg salbutamol + 150 mg theophylline + 1% HPMC + 40% PEG400	Salbutamol flux across human skin	13.36 ± 1.02 μg/cm^2^ h	[38]
	10 mg terbutaline in bilosomes	Cumulative amount of terbutaline permeated across mouse skin over 24 h	0.3% chitosan at high lipid level: 333.81 ± 10.35 μg/cm^2^	[26]
	Terbutaline in aqueous isopropanol	Flux across human skin	Up to 100 μg/cm^2^ h in split-thickness skin and 26 μg/cm^2^ h in dermis	[39]
	1.25 mg terbutaline + PVA + PVP + 35% PG	Flux across guinea pig skin	Up to 7.9076 μg/cm^2^ h	[40]
	4 mg terbutaline + 2% HPMC + 40% PEG + 4% IPM	Flux across human skin	5.10 ± 0.21 μg/cm^2^ h	[28]
	3.65 mg terbutaline + Eudragit RL-100/RS-100 (2/1)	Flux across mouse or human skin	Mouse skin: up to 63.57 μg/cm^2^ h with IPM Human skin: up to 59.13 μg/cm^2^ h with IPM	[41]
	Terbutaline + PG	Flux across rabbit, guinea pig, or human skin	Rabbit skin No azone: 8.3 ± 2.3 μg/cm^2^ h With 3% azone: 28.5 ± 6.2 μg/cm^2^ h	[42]
			Guinea pig skin No azone: 7.7 ± 1.9 μg/cm^2^ h With 3% azone: 56.1 ± 6.6 μg/cm^2^ h	
			Human skin No azone: 0.6 ± 0.1 μg/cm^2^ h With 3% azone: 3.6 ± 0.8 μg/cm^2^ h	
*In vivo*	16% salbutamol + 71.9% silicone + 10% n-dodecanol + 1.75% glycerol + 0.35% hexanol	Serum concentration (Rhesus monkey)	Single layer: 44.6 ± 16.42 ng/mL Double layer: 62.53 ± 7.98 ng/mL	[27, 34]
		Bioavailability (Rhesus monkey)	Single layer: 20.58 ± 7.42% Double layer: 19.88 ± 1.89%	
		Normalized amount absorbed (Rhesus monkey)	Single layer: 0.206 ± 0.074 mg/day/dose Double layer: 0.211 ± 0.015 mg/day/dose	
	2% salbutamol-loaded cubosomal gel	Cmax (Wistar rat)	21.56 ± 5.29 ng/mL	[25]
	2.5 mg salbutamol + 150 mg theophylline + 1% HPMC + 40% PEG400	Cmax (Human)	4.6 ± 1.3 ng/mL	[38]
		AUC_0-24_ (human)	86.4 ± 8.7 ng*h/mL	
	10 mg terbutaline in bilosomes	Cmax (rat)	Oral: 77.41 ± 5.33 ng/mL TBN-CTS-BLS gel: 57.28 ± 5.37 ng/mL	[26]
		AUC_0-24_ (rat)	Oral: 167.99 ± 7.20 ng*h/mL TBN-CTS-BLS gel: 384.49 ± 21.30 ng*h/mL	
		Bioavailability relative to oral	TBN-CTS-BLS gel: 233.62% in rats	

AUC_0-24_ = Area under the plasma-concentration curve from 0 to 24 h; Cmax = Maximum plasma drug concentration; Css = Steady-state plasma drug concentration; DMAPMA = N-[3-(dimethylamino)propyl]methacrylamide; EtOH = Ethanol; HEMA = 2-hydroxyethyl methacrylate; HPC = Hydroxylpropylcellulose; HPMC = Hydroxypropyl-methylcellulose; IPM = Isopropyl myristate; MO = DL-α-Monoolein; PEG400 = Polyethylene glycol; PG = Propylene glycol; PVA = Polyvinyl alcohol; PVP = Polyvinyl pyrrolidone; Q_24_ = Cumulative amount permeated in 24 h; TBN-CTS-BLS gel = Terbutaline-chitosan-bilosomal gel.

#### Salbutamol

Also known as albuterol, salbutamol is commonly used for acute asthma reactions [1, 5, 7, 8, 22, 23]. Serum concentrations ranging from 4 to 8 ng/mL are ideal for treating bronchoconstriction (higher concentrations up to 33 ng/mL are targeted for other indications such as uterine relaxation for preterm labor) [34]. Salbutamol was found to have passive skin permeability as early as 1989 and has since shown promise with transdermal delivery for not only asthma but also as a model drug for developing innovative transdermal formulations [24, 25, 27, 29–31, 33, 35–38, 43–45]. For asthma applications, salbutamol has been successfully loaded into polymer-based films, cubic nanoparticles, and ethanol-containing liposomes called ethosomes [24, 25, 27, 29, 37, 38].

Polymeric film formulations incorporating hydroxypropyl methylcellulose (HPMC) and silicone sustained controlled salbutamol skin permeation for up to 30 h, reaching *in vivo* concentrations in the ng/mL range in both monkeys and humans [27, 29, 34]. Double layer silicone pads applied to shaved chests of monkeys reached serum concentrations as high as 62 ng/mL in 24 h and steadily declined after patch removal, whereas intravenous administration reached a similar serum concentration but dropped off in under 10 h [27]. Salbutamol in an HPMC gel reached a steady-state serum concentration of 2.87 ng/mL in humans which was maintained until patch removal at 24 h, after which a reservoir effect sustained serum concentrations for 2 h before starting to decrease at 26 h [29]. Additional *in vitro* studies with poly(hydroxyethyl methacrylate)-based co-polymers across excised mouse skin achieved up to 30% cumulative permeation with a co-polymer formulation of 2-hydroxyethyl methacrylate with N-[3-(dimethylamino)propyl]methacrylamide in a 50/50 ratio [35]. Eudragit polymers with added plasticizers or enhancers permitted ∼4 mg of 6 mg loaded salbutamol to permeate across rabbit skin *in vitro* over 24 h [24].

Salbutamol loaded into various carriers has been investigated to achieve improved bioavailability and a sustained release profile. Encapsulation of salbutamol within DL-α-monoolein (MO)/Pluronic F127 cubosomal nanoparticles achieved a biphasic release profile of an initial burst within 30 min and sustained salbutamol permeation through rat skin *ex vivo* over 72 h [25]. Subsequent *in vivo* testing with rats demonstrated higher serum concentrations and a longer release (up to 72 h) from the cubosomal gel vs. control gel, resulting in an 8.62-fold increase in bioavailability [25]. In another study, salbutamol loaded into ethosomes also achieved a sustained release delivery profile, with significantly greater drug permeation across mouse skin *in vitro* over 24 h compared to the liposomal formulation [37].

Iontophoresis (a physical permeation enhancement) increased salbutamol permeation across skin from a liquid crystalline vehicle, reaching delivery rates up to 1.9 mg/min vs. 0.3 mg/min of passive permeation through the skin [31, 32]. Transdermal salbutamol flux enhancement with iontophoresis varies based on ionization of the drug and whether ion-pairs form (in the skin or the formulation) that neutralize the charge, as well as the frequency and pulsing of the iontophoretic current [31, 36].

#### Terbutaline

Despite its low skin permeability, terbutaline has also been investigated for transdermal delivery, and permeation is enhanced when formulated with isopropanol and permeation enhancers [26, 28, 39–42, 46]. Addition of isopropyl myristate (IPM) produced flux values of 63 μg/cm^2^ h and 59 μg/cm^2^ h compared to 55 μg/cm^2^ h and 50 μg/cm^2^ h with non-enhanced control in mouse skin and in human skin, respectively [41]. In another case, when formulated with IPM, terbutaline flux reached 5.1 μg/cm^2^ h [28]. The 10-fold difference in flux between these studies can readily be explained by differences in terbutaline concentration in the formulations. The formulation developed by Panigrahi, Pattnaik, and Ghosal [41] contained 3.65 mg/cm^2^ of terbutaline in the films, whereas the formulations by Murthy and Hiremath [28] contained only 0.4 mg/cm^2^. Regardless of concentration, these transdermal terbutaline formulations provided sustained delivery for up to 72 h [26, 28, 40–42].

#### Acute episodes

Based on disease-specific considerations (e.g., difficulty inhaling during an acute attack) alternate delivery routes such as transdermal may be useful even for acute attacks. To be realistic as an acute standalone rescue treatment, transdermal SABA delivery would require very rapid absorption. This is possible with some physical enhancement techniques (iontophoresis, microneedles) but has not been specifically explored in asthma. Inhalation will likely remain gold standard first-line therapy, but the pharmacokinetic profile from transdermal delivery could provide specific advantages, such as preventing further deterioration during an acute attack and/or protecting against further attacks–supporting a potential role of transdermal SABAs as an adjunct therapy in acute settings.

### Long-acting β2-agonists (LABAs)

LABAs are prescribed as maintenance therapies for chronic asthma and can sustain bronchodilation for up to 12 h [7, 8], and are often paired with ICSs for inhaled administration [11, 22, 23]. Endpoints from transdermal LABA studies can be found in Table 2.

**TABLE 2:** Long-acting β2-agonists.

Experimental technique	Drug and formulation	Endpoint	Results	Ref.
*In vitro*/*ex vivo*	Tulobuterol commercial patch	Percent tulobuterol released	2 mg dose: 40% released over 24 h from 2 mg/day patch	[54]
*In vivo* (Human)	Tulobuterol commercial patch	Serum tulobuterol concentration, reported as Css or Cmax	Css: 1.82 ng/mL (achieved with 2 mg/day patch)	[48, 51, 54, 64]
			1 mg/day patch: Cmax: 1.39 ± 0.22 ng/mL AUC_0-t_: 29.03 ± 4.38 ng*h/mL	
			Cmax 2 mg dose: 1.4 ± 0.1 ng/mL 4 mg dose: 3.3 ± 0.7 ng/mL 6 mg dose: 4.3 ± 1.0 ng/mL	
		PEF	Increased from 178.3 ± 13.3 to 246.7 ± 20.6 L/min over 11 h of administration in pediatric patients	[54]
			50 L/min increase in the evening after 16 weeks and 60 L/min increase in the morning after 10 weeks in adults	
	Tulobuterol commercial patch + ICS	PEF	372.5 ± 21.7 L/min to 411.2 ± 31.1 L/min	[57]
			13.3 L/min increase	[62]
		Change in %FEV_1_	−1.98 ± 10.48%	[53]
			68.7 ± 1.6% (baseline) to 70.4 ± 1.7% (tulobuterol + ICS)	[62]
		Change in %FVC	96.4 ± 3.67% (ICS) to 99.0 ± 3.61% (tulobuterol + ICS)	[57]
	Tulobuterol commercial patch + inhaled tiotropium	Change in FVC	3.27 ± 0.62 to 3.59 ± 0.64 L	[58]
			2.61 ± 0.12 L to 2.92 ± 0.12 L	[59]
		Change in FEV_1_	1.41 ± 0.47 to 1.63 ± 0.45 L	[58]
			1.16 ± 0.09 L to 1.32 ± 0.09 L	[59]

AUC_0-t_ = Area under the plasma-concentration time curve from 0 to the time of the last quantifiable concentration; Cmax = Maximum plasma drug concentration; Css = Steady-state plasma drug concentration; ICS = Inhaled corticosteroid; FEV_1_ = Forced expiratory volume in 1 s; %FEV_1_ = Change in percent predicted forced expiratory volume in 1 s; FVC = Forced vital capacity; %FVC = Change in percent predicted forced vital capacity; PEF = Peak expiratory flow rate.

#### Tulobuterol

As the first asthma therapy formulated in a transdermal patch, tulobuterol established feasibility of transdermal delivery systems for respiratory therapies [47–51]. First available in Japan in 1988, it is now used in Japan, China, and Korea. The patch is applied once daily, typically at bedtime, and worn for 24 h to provide controlled tulobuterol delivery - achieving peak effect in the morning during the “morning dip” when respiratory function is typically lower [48, 52, 53]. The patch provides sustained delivery with more consistent blood concentrations than oral tablets [50, 51, 54–56]. Oral tulobuterol is associated with adverse effects such as tremors and tachycardia related to high tulobuterol blood concentrations; however, these effects are reduced with the tulobuterol patch because of a controlled release profile that avoids high blood concentrations [49, 50, 54]. The 1 and 2 mg/day patches both significantly improve forced (peak) expiratory flow (PEF) [50].

#### Combination therapies with inhaled corticosteroids

The tulobuterol patch has also been investigated as add-on treatment for patients using ICS. The patch + ICS combination significantly improved PEF compared to patients using ICS alone and demonstrated comparable improvements as other add-on treatments such as pranlukast and slow-release theophylline [57]. Beyond asthma, the tulobuterol patch has been paired with inhaled tiotropium (a long-acting muscarinic antagonist) for treatment of chronic obstructive pulmonary disease [58–60]. The combination resulted in significantly improved pulmonary function compared to either treatment separately [60]. While this pairing of the tulobuterol patch with a long-acting muscarinic antagonist has not been explored as a treatment for asthma, its effectiveness in another pulmonary disorder is encouraging.

Additionally, effectiveness of the tulobuterol patch has been compared to other commonly used LABAs, including salmeterol and formoterol, as add-on therapies to ICS treatment [54–56, 61]. While tulobuterol in this combination approach was effective at improving symptoms, inhaled salmeterol exhibited greater clinical efficacy than transdermal tulobuterol when included as an add-on therapy to ICS treatment. Salmeterol + ICS consistently resulted in significantly greater changes in forced expiratory volume in one second (FEV_1_), PEF, inspiratory capacity (IC), and forced vital capacity (FVC) compared to baseline measurements [52, 62–64].

Inhaled formoterol in combination with ICS did not significantly improve FEV_1_ or FVC compared to the tulobuterol patch + ICS combination, but it did improve resonant frequency significantly more than the tulobuterol patch, indicating an improvement in airway resistance [53, 65]. While the efficacy of the tulobuterol patch was generally lower than the inhaled LABAs, patients had higher compliance rates with the patch than the inhaled drugs [66]. Overall, these studies provide evidence that transdermal LABAs could be useful maintenance therapies in addition to ICS because of the improved delivery profile and patient compliance [67, 68]. This could be particularly advantageous for specialized populations including children or older patients [69].

#### Formoterol

Formoterol has also been investigated for transdermal applications [70]. Various excipients were screened for their ability to enhance formoterol permeation across excised rat abdominal skin. Cineole, isopropyl myristate (IPM), and l-menthol were subsequently incorporated into mixed solvent systems with N-methyl-2-pyrrolidone (NMP) to aid in drug solubility. For each mixed solvent system, 54 μg of formoterol in 135 μL of the mixed solvent were applied to rat skin with a diffusion area of 0.95 cm^2^. Formoterol in NMP as a single solvent system permeated 2.4 μg/cm^2^ over 24 h, which was not significantly higher than the saline control of 1.2 μg/cm^2^. The addition of cineole, IPM, and l-menthol as co-solvents significantly increased formoterol permeation to ∼50 μg/cm^2^ over 24 h for solvent ratios of 50/50 of cineole/NMP, 60/40 of l-menthol/NMP, and 40/60 of IPM/NMP [70].

## Discussion

Here we reviewed potential benefits of transdermal SABA and LABA delivery, providing a high-level overview of reports extending back nearly 40 years. Transdermal β2-agonist delivery offers a novel approach to asthma management but has received minimal attention. Ongoing challenges include limited consideration for pulmonary conditions, unfavorable physicochemical properties of many β2-agonists (specifically, wide variation in hydrophilicity), and high dose requirements that are difficult with conventional transdermal dosage forms.

These challenges may be addressable with advances in physical enhancement techniques such as microneedles and iontophoresis. Transdermal delivery of hydrophilic and ionized compounds is now readily achievable [71–73], as is delivery of larger doses (up to 33 mg) [74, 75]. These advances could minimize limitations that would directly limit applicability of transdermal dosage forms for drugs like terbutaline that require higher doses [76, 77]. Sustained transdermal delivery profiles would translate to improved pharmacokinetics, preventing troughs in plasma drug concentrations and improving efficacy and long-term disease management [78, 79]. Microneedles permit rapid dermal absorption (beginning in <5 min) of molecules of highly varied physicochemical properties [75, 80–83], which opens the possibility of transdermal β2-agonists as an adjunct in rescue therapy – primarily to quickly achieve and maintain suitable plasma drug levels after the inhaled therapy has brought an acute attack under control.

In many patients (especially those with severe asthma), additional therapies beyond β2-agonists are added to treatment because outcomes may still not be optimized. More recently this has included monoclonal antibodies [9, 10]. Part 2 of this mini-review series highlights studies of transdermal delivery of other asthma and allergy treatments including leukotriene modifiers, mast cell stabilizers, muscarinic antagonists, adjunct therapies, and biologics.
